# The *Him* Gene Reveals a Balance of Inputs Controlling Muscle Differentiation in *Drosophila*

**DOI:** 10.1016/j.cub.2007.07.039

**Published:** 2007-08-21

**Authors:** David Liotta, Jun Han, Stuart Elgar, Clare Garvey, Zhe Han, Michael V. Taylor

**Affiliations:** 1 Cardiff School of Biosciences, Cardiff University Main Building, Cardiff CF10 3TL, United Kingdom; 2 Department of Zoology, University of Cambridge, Cambridge CB2 3EJ, United Kingdom; 3 Department of Molecular Biology, University of Texas Southwestern Medical Center, Dallas, Texas 75390

**Keywords:** DEVBIO

## Abstract

Tissue development requires the controlled regulation of cell-differentiation programs. In muscle, the Mef2 transcription factor binds to and activates the expression of many genes and has a major positive role in the orchestration of differentiation [1–4]. However, little is known about how Mef2 activity is regulated in vivo during development. Here, we characterize a gene, *Holes in muscle* (*Him*), which our results indicate is part of this control in *Drosophila*. *Him* expression rapidly declines as embryonic muscle differentiates, and consistent with this, *Him* overexpression inhibits muscle differentiation. This inhibitory effect is suppressed by *mef2*, implicating *Him* in the *mef2* pathway. We then found that *Him* downregulates the transcriptional activity of Mef2 in both cell culture and in vivo. Furthermore, Him protein binds Groucho, a conserved, transcriptional corepressor, through a WRPW motif and requires this motif and *groucho* function to inhibit both muscle differentiation and Mef2 activity during development. Together, our results identify a mechanism that can inhibit muscle differentiation in vivo. We conclude that a balance of positive and negative inputs, including Mef2, Him, and Groucho, controls muscle differentiation during *Drosophila* development and suggest that one outcome is to hold developing muscle cells in a state with differentiation genes poised to be expressed.

## Results and Discussion

Analysis of *mef2* function during *Drosophila* muscle development has shown that a major aspect of its role is in the differentiation pathway downstream of the genes that specify muscle [5–7]. However, Mef2 protein expression precedes muscle differentiation [1]. It is first expressed in the mesoderm at gastrulation, approximately 3 hr after egg laying (AEL) [6]. This is approximately 7 hr before the activation at stage 13 (10 hr AEL) of the expression of many known Mef2 target genes, e.g., *Mhc*, *Mlc1*, and *wupA* ([4, 8]; data not shown). This delay implies that the activity of Mef2 is restrained and that other regulatory proteins operate in the control of muscle differentiation during this period. However, little is known about these other proteins nor about how the gene expression at stage 13 is coordinated. Here, we address these unanswered questions through an analysis of the *Him* gene in muscle differentiation. *Him* was described in a computational screen [9], and we isolated it separately in an expression screen [10], but its function has not previously been analyzed. We demonstrate that it is an inhibitor of Mef2 activity and muscle differentiation, and on the basis of this phenotype, we call it *Holes in muscle* (*Him*).

**Figure 1:**
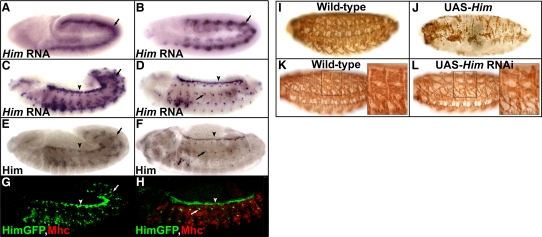
*Him* Expression and Function in Muscle Development (A–H) *Him* expression decreases as muscle differentiates. In situ hybridization for *Him* RNA (A–D), immunostaining for Him protein (E and F), and double immunostaining for a Him-GFP fusion protein (green) and Myosin heavy chain (Mhc) protein (red) (G and H) are shown. The anterior is shown to the left, and dorsal side is shown uppermost, here and in all other figures. Stage 9 and 11 embryos showing that *Him* is initially expressed widely in the mesoderm (arrow) are shown in (A) and (B), respectively. Stage 12 embryos (∼8 hr 20 AEL) showing *Him* expression in somatic muscle precursors (arrow) and heart precursors (arrowhead) are shown in (C), (E), and (G). Stage 13 embryos (∼9 hr 50 AEL) showing absence of *Him* expression in developing somatic muscle and continued expression in adult muscle precursors (arrow) and developing heart (arrowhead) are shown in (D), (F), and (H). (I and J) *Him* inhibits muscle differentiation in vivo. An immunostain for Mhc of stage 17 embryos with *UAS-Him* expression driven in the developing mesoderm by *twi-Gal4;twi-Gal4* at 25°C shows that *Him* inhibits the terminal differentiated muscle phenotype. (I) shows the wild-type, and (J) shows *Him* overexpression. A representative example of the phenotype is shown. (K and L) *Him* knockdown embryos have abnormal muscle differentiation. An immunostain for Mhc of stage 17 embryos with *UAS-Him RNAi* driven by *twi-Gal4;twi-Gal4* at 25°C shows that *Him* is required for correct muscle differentiation. (K) shows the wild-type, and (L) shows *Him* knockdown. The *Him* knockdown muscle phenotype was assayed as described in Experimental Procedures. Approximately one-third of the embryos had six or more muscles per hemisegment with abnormal morphology. (L) shows a representative example of this phenotype in which most of the dorsal muscles are misshapen and are frequently thinner than the wild-type. Approximately one-third had a weaker phenotype, and approximately one-third had no apparent phenotype.

*Him* has a striking, transient pattern of expression during *Drosophila* embryogenesis. It is first expressed during stage 9 broadly in the mesoderm (Figure 1A). This expression then refines, and at stage 12 it is specifically expressed in the precursors of the somatic musculature and of the heart (Figure 1C). *Him* expression then rapidly declines in the somatic mesoderm, such that in 90 min it has disappeared from the differentiating somatic muscle (stage 13, Figure 1D). However, it persists in the adult muscle precursors (AMPs), which are set aside in the somatic mesoderm and which remain undifferentiated at this stage, and also in the developing heart. Him protein expression closely resembles that of *Him* RNA (Figures 1E and 1F). The disappearance of Him coincides with the expression of Myosin, a classic marker of muscle differentiation. Double labeling with a Him-GFP fusion gene (see Experimental Procedures in the Supplemental Data available online) demonstrates that Myosin is expressed only after Him disappears from the developing muscle (Figures 1G and 1H). The expression of Him in the progenitors of the somatic muscle and its disappearance from differentiating muscle are consistent with a role for *Him* as an inhibitor of muscle differentiation.

### *Him* Inhibits Muscle Differentiation In Vivo

To test whether *Him* is an inhibitor of muscle differentiation, we overexpressed it in the developing mesoderm by using the Gal4/UAS system [11]. This induced a dramatic reduction in the number of Myosin-expressing cells and thereby produced large gaps or holes in the musculature (Figures 1I and 1J). We then asked when in muscle development *Him* has this effect. We found that up to stage 13 (10 hr AEL) muscle development proceeds similarly to that of the wild-type. At this stage, developing muscles are seen in the wild-type as small syncytia, which express founder cell markers, e.g., Kruppel [12], and Mef2, surrounded by Mef2-expressing myoblasts. When *Him* is overexpressed, the expression of these markers is similar (Figures S1A–S1D). Subsequently, immunostaining for Mef2 reveals disrupted differentiation at stage 15, and there is increased cell death at stage 16 (Figures S1E–S1H). Together, these findings demonstrate that *Him* inhibits the differentiation phase, of muscle development, that occurs from stage 13 onward and that produces the morphologically distinct muscles of the functional musculature by the end of embryogenesis. To explore this function further, we knocked down *Him* by using RNAi from a splice-activated UAS hairpin vector (see Experimental Procedures and Figure S2). Although the musculature develops similarly to that of the wild-type, in the knockdown there is impaired muscle differentiation as revealed by disrupted muscle morphology (Figures 1K and 1L).

### *Him* Genetically Interacts with *mef2*

**Figure 2:**
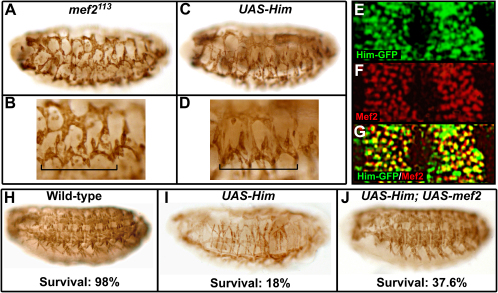
*Him* Genetically Interacts with *mef2* (A–D) An immunostain for Mhc at stage 17 shows that overexpression of *Him*, from *24B-Gal4* × *UAS-Him* at 25°C (C and D), phenocopies the *mef2^113^* hypomorph (A and B). (E–G) Him is expressed in somatic muscle precursors with Mef2. Confocal microscopy of developing somatic muscle in two hemisegments at stage 12 showing Him-GFP (E), Mef2 (F), and merge (G) is shown. (H–J) *mef2* suppresses the muscle phenotype and lethality induced by *Him* overexpression from *twi-Gal4;twi-Gal4* × *UAS-Him* at 18°C. An immunostain for Mhc at stage 17 shows the wild-type differentiated muscle pattern (H), the inhibited muscle development from *Him* overexpression (I), and the suppression of this phenotype by coexpression of *UAS-mef2* with *UAS-Him* (J). Each panel shows a representative phenotype for each condition and the percentage of survival until adulthood. The number of wild-type muscles per three hemisegments in *UAS-Him* with *UAS-mef2* was 61.0 ± 19.0 (mean ± SD, n = 51 embryos) and significantly higher than in *UAS-Him* alone, 39.1 ± 20.3 (mean ± SD, n = 50 embryos) (p < 0.001, two-sample t test).

We found that the overexpression of *Him* during muscle development phenocopies the *mef2^113^* hypomorphic allele (Figures 2A–2D). Development of the musculature is inhibited similarly, and many of the residual muscles have a similar, abnormal morphology. This suggests that the two genes function in a common pathway. Consistent with this, Him and Mef2 are coexpressed in somatic muscle progenitors at stage 12, prior to the activation of muscle-differentiation markers such as Myosin (Figures 2E–2G). To test whether *Him* and *mef2* genetically interact, we overexpressed both genes together. Strikingly, the inhibition of muscle differentiation caused by *Him* is rescued toward the wild-type by *mef2* (Figures 2H–2J). Furthermore, overexpression of *Him* alone induces lethality, and under the conditions of this experiment only 18% survive. This lethality is suppressed by *mef2*, and there are more than twice as many survivors. Together, our phenotypic analysis and genetic interaction findings indicate that *Him* functions in the Mef2 pathway that controls muscle differentiation.

### *Him* Requires *groucho* to Mediate Its Function

**Figure 3:**
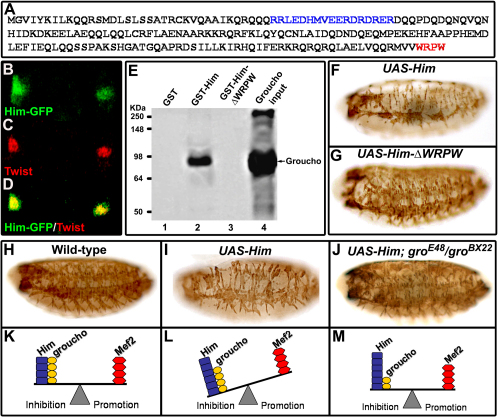
*Him* Requires *gro* to Mediate Its Action (A) Him predicted protein with a putative NLS (see Experimental Procedures) and a C-terminal WRPW motif, highlighted in blue and red, respectively. (B–D) Him is a predominantly nuclear protein. Confocal microscopy of two AMPs showing Him-GFP (B), the nuclear Twist protein (C), and merge (D) is shown. (E) Autoradiograph of a protein gel of a “pulldown” assay showing that GST-Him (lane 2), but not GST-HimΔWRPW (lane 3) or GST alone (lane 1), binds to the input radiolabelled Groucho (lane 4). (F and G) Him requires its WRPW motif to inhibit muscle differentiation. An immunostain for Mhc at stage 17 shows that expression of *UAS-HimΔWRPW* driven in the developing mesoderm by *twi-Gal4;twi-Gal4* at 25°C produces no dramatic loss of muscles (G), in contrast to full-length *UAS-Him* (F). (H–J) *Him* requires *gro* function to inhibit muscle differentiation. *gro* suppresses the muscle phenotype induced by *Him* overexpression from *twist-Gal4* × *UAS-Him* at 25°C. An immunostain for Mhc at stage 17 shows the wild-type differentiated muscle pattern (H), the inhibited muscle development from *Him* overexpression (I), and the suppression of this phenotype in a *gro^E48^*/*gro^BX22^* mutant background (J). Each panel shows a representative phenotype for each condition. The number of wild-type muscles per three hemisegments in *UAS-Him* in a *groucho* background was 61.0 ± 16.6 (mean ± SD, n = 45 embryos) and significantly higher than in *UAS-Him* alone, 35.3 ± 16.7 (mean ± SD, n = 52 embryos) (p < 0.001, two-sample t test). (K–M) Schematic representation of the balance between promoting and restraining influences controlling muscle differentiation illustrated by these experiments.

The Him protein sequence includes a putative bipartite nuclear localization signal (NLS) (Figure 3A). Consistent with this, colocalization with the transcription factor Twist in the AMP nuclei shows that Him is predominantly nuclear (Figures 3B–3D). The Him protein also has a WRPW motif at its C terminus (Figure 3A), as noted previously [9]. This tetrapeptide in this position is found in the Hairy group of transcriptional repressors and mediates their interaction with the corepressor Groucho (Gro) [13, 14]. We used a pulldown assay to show that Him can also bind Gro (Figure 3E). Moreover, this interaction requires the WRPW motif because Him with the WRPW motif deleted (HimΔWRPW) cannot bind Gro. To investigate the importance of the WRPW for Him function, we then overexpressed HimΔWRPW in embryos and found there was no dramatic loss of muscles, in contrast to the effect of full-length Him (Figures 3F and 3G). Together, these results show that Him can bind Gro through its WRPW tetrapeptide and that this motif is required to inhibit muscle differentiation.

We then investigated the significance of the Him/Gro interaction in vivo during embryonic muscle development by overexpressing *Him* in a *gro* mutant background. Strikingly, the loss of *gro* function suppresses the inhibitory effect of *Him* (Figures 3H–3J), showing that Him requires *gro* to inhibit muscle differentiation. This result, together with our finding that *mef2* can suppress the inhibitory effect of *Him* (Figure 2), indicates that *Drosophila* muscle differentiation in vivo is controlled by a balance between the activities of Him and Gro on the one hand and Mef2 on the other (Figures 3K–3M). The effect of overexpression of Him can be balanced by a reduction in Gro or by an increase in Mef2 (Figures 2H–2J).

### *Him* Downregulates Mef2 Activity

**Figure 4:**
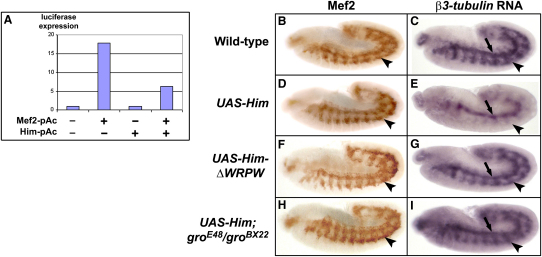
*Him* Downregulates Mef2 Activity (A) *Him* inhibits Mef2 activity in cell culture. In transfected S2 cells, which contain significant levels of Gro [21], a Mef2-responsive miR1-luciferase reporter is activated by Mef2. This effect is inhibited by cotransfection with *Him*. (B–I) *Him* inhibits Mef2 activity in vivo during muscle development. Mef2 protein is visualized by an immunostain of stage 12 embryos (B, D, F, and H). Somatic mesoderm expression (arrowhead) is indicated. Expression of a Mef2 target in the developing somatic muscle, *β3-tubulin*, is visualized by in situ hybridization of stage 12 embryos (C, E, G, and I). Somatic mesoderm (arrowhead) and visceral mesoderm (arrow) expression are indicated. (B) and (C) show the wild-type; (D) and (E) show that expression of *UAS-Him* driven by *twi-Gal4; twi-Gal4* at 25°C does not affect Mef2 protein expression, but dramatically downregulates *β3-tubulin* expression in the somatic mesoderm. Expression of *β3-tubulin* is unaffected in the visceral mesoderm, where it is not a Mef2 target [15], but where *twi-Gal4; twi-Gal4* drives *Him* expression at this stage (data not shown). Downregulation of *β3-tubulin* expression is not seen if either the Him C-terminal WRPW motif is deleted (F and G), or the experiment is in a *gro^E48^*/*gro^BX22^* mutant background (H and I).

To further investigate the mechanism of action of Him, we asked whether *Him* could inhibit Mef2 activity in cell culture in a direct Mef2-dependent gene-expression assay. When *mef2* was transfected into S2 cells, it stimulated the expression of a Mef2-responsive luciferase reporter, and this effect was inhibited by cotransfection with *Him* (Figure 4A). We then tested whether *Him* could also inhibit Mef2 activity in the context of muscle development. We analyzed the effect of *Him* overexpression on the expression of Mef2 and of *β3-tubulin*, which is a direct Mef2 target gene in somatic muscle [15]. *β3-tubulin* expression is strongly reduced in the somatic mesoderm, whereas Mef2 protein expression is similar to that of the wild-type (Figures 4B–4E). This indicates that *Him* can downregulate Mef2 activity in vivo during embryonic development. We could further show that Him with the Gro-interacting WRPW motif deleted does not affect *β3-tubulin* expression (Figures 4F and 4G), nor does full-length Him in a *groucho* mutant background (Figures 4H and 4I).

Taken together, our combination of in vitro and in vivo assays (Figures 3 and 4) reveals key features of Him's mechanism of action. They demonstrate that Him is found in the nucleus and requires its Gro-binding WRPW motif and *gro* function to inhibit both Mef2 activity and muscle differentiation during development. The previously characterized *Drosophila* proteins that have a C-terminal Gro-interacting WRPW motif are the Hairy group of HLH domain DNA-binding transcriptional repressors [14]. However, Him is novel and does not have an HLH domain (see Experimental Procedures), suggesting that it does not bind DNA directly. Its mechanism of action may have parallels with Ripply1, which functions in vertebrate somitogenesis [16]. Ripply1 also appears not to be an HLH protein and yet contains a functional Gro-interacting WRPW motif, although in this case near the N-terminus of the protein. Like Ripply1, Him may be part of a transcriptional-repressor protein complex. The precise mechanism by which Him targets Mef2 awaits analysis of this putative complex and the protein partners within it.

### Conclusions

Despite considerable progress, much remains to be learned about the regulation of muscle differentiation during animal development. Although studies in cell culture indicate that this control might include negative mechanisms [17–19], little is known about the identity and mode of action of specific molecules that inhibit muscle differentiation in vivo during development. Here, we have identified and analyzed one mechanism that involves *Him* and that can do this through targeting Mef2, the key positive regulator of muscle differentiation, and downregulating its transcriptional activity. This inhibitory action of Him, coupled to its transient expression in developing muscle cells, is an explanation for the observation that Mef2 is present significantly before overt differentiation. It also offers an explanation for how a burst of expression of many Mef2 target genes at a specific phase (stage 13) of the differentiation program is coordinated. We suggest that the rapid decrease in the expression of Him will lead to a concomitant increase in the activity of Mef2 and the ability to activate a cohort of these genes. Further studies will determine whether this will link to a recent report that the ability of Mef2 to bind DNA is temporally regulated [4].

Our results also indicate that the inhibition of Mef2 activity by endogenous levels of Him is incomplete prior to stage 13. Thus, in normal muscle development, the Mef2 target gene *β3-tubulin* is expressed at stage 12, even though we find that overexpression of Him can downregulate its expression then. This implies that in the wild-type embryo, there is some Mef2 activity at stage 12, and such activity is sufficient for *β3-tubulin* expression. This is consistent with other work that indicates that Mef2 regulates some gene expression at this stage and earlier [3, 4, 10] and suggests that Him can provide one level of control of Mef2 activity during the muscle-differentiation program. Taken together, our results move the molecular analysis of muscle differentiation on from a simple model in which the key events are expression of pivotal positive regulators, for example Mef2. Rather they indicate that muscle differentiation in vivo is controlled by a balance of positive and negative regulators, including Him, Gro, and Mef2, that governs whether muscle precursors differentiate. In this model, one can think of Him and Gro as part of a mechanism holding the cells in a committed, but undifferentiated, state in which a cohort of muscle-differentiation genes is poised to be expressed. This might be a widespread strategy for coordinated gene expression in cell-differentiation programs. For example, it can be compared with melanocyte stem cell differentiation, where cells are primed to rapidly express terminal differentiation markers once Pax3/Groucho-mediated repression is relieved [20].

## Supplemental Data

Document S1. Experimental Procedures and Two Figures
