# Supplementary material for: The Him Gene Reveals a Balance of Inputs Controlling Muscle Differentiation in Drosophila
Source: Curr Biol. 2007 Aug 21;17(16):1409–13. doi: 10.1016/j.cub.2007.07.039 (PMC1955682; doi:10.1016/j.cub.2007.07.039)
Supplement: Document S1. Experimental Procedures and Two Figures [file mmc1.pdf]

Supplemental Data

The *Him* Gene Reveals a Balance of Inputs Controlling Muscle Differentiation in *Drosophila*

David Liotta, Jun Han, Stuart Elgar, Clare Garvey,
Zhe Han, and Michael V. Taylor

Supplemental Experimental Procedures

In Situ Hybridization, Immunohistochemistry, and Acridine Orange Assay

In situ hybridizations with Digoxigenin-labeled RNA probes were as described previously [S1]. The DNA template for the *Him* probe was a cDNA in pBluescript II KS (−) isolated from a subtracted library [S1], and the DNA template for *β3-tubulin* was a *β3-tubulin* cDNA (a gift from R.Renkawitz-Pohl) subcloned into pBluescript II KS (+). Embryos were mounted in 80% glycerol. Acridine orange staining was essentially as described in Sullivan et al. [S2]. Single-antibody staining was as described in Ruiz-Gomez and Ghysen [S3] with the following primary antibodies: rabbit anti-Mhc (1:500, gift from D.Kiehart), guinea pig anti-Kruppel (1:1000, gift from D.Kosman), rabbit anti-Mef2 (1:1000, gift from B.Paterson), and guinea pig anti-Him (1:1500). Embryos were mounted either in an acetone: araldite mixture (1:1 ratio) or in 80% glycerol. In each case, embryos were viewed with a Zeiss Axioskop microscope, and images were captured with Axiovision software. Fluorescent double stainings were essentially as in Kaltschmidt et al. [S4] with the following primary antibodies: mouse anti-GFP (1:3000, Sigma) plus one of rabbit anti-Mhc (1:500), rabbit anti-Mef2 (1:1000), or rabbit anti-Twist (1:5000, gift from S. Roth). Embryos were mounted in Vectashield fluorescent mounting medium (Vector laboratories) and visualized on a Leica confocal microscope; images were processed with the Leica software. The indicated times of different stages of embryogenesis are for development at 25°C.

Generation of Transgenic Lines

We made *UAS-Him* and *UAS-Him-ΔWRPW* by subcloning from *Him* cDNA in pBluescript II KS (−) [S1] into pUAST [S5] as a restriction fragment and as a PCR-amplified fragment for removal of the C-terminal WRPW, respectively. All PCR-generated DNA fragments used in this project were sequence verified, and the primer sequences are available on request. We made *UAS-Him-RNAi* by cloning the complete PCR-amplified *Him* coding sequence into pGEM-T (Promega) and subcloning the sequence into pJM1084 (a splice-activated UAS hairpin vector [S6]) on both sides of the intronic spacer (antisense 5′ of the intron, sense 3′ of the intron). The Him-GFP fusion gene construct comprised approximately 3.8 kb of upstream genomic sequence, the mGFP6 variant [S7] fused via a -SSSS- linker to the N-terminus of the Him coding sequence, and the *Him* 3′UTR. The GFP sequence was inserted by standard PCR procedures. Transgenic lines for all constructs were generated by injection of *yw* embryos according to published procedures [S8], and stocks homozygous for each construct were made. For each construct, a minimum of three independent transgenic lines was tested.

Drosophila Stocks and Crosses

The following stocks maintained on standard culture medium were used: *Oregon R*, *UAS-Him*, *UAS-Him-RNAi*, *UAS-Him-ΔWRPW*, *Him-GFP*, *UAS-mef2* [S9], *mef2*^{113} [S10], *gro*^{E48} [S11], and *gro*^{BX22} [S11]. Expression of the *UAS* constructs was achieved with the following Gal4 lines at the temperature indicated in the text: *twi-Gal4;twi-Gal4* [S12], *24B-Gal4* [S5], and *twi-Gal4* (made from *twi-Gal4;mef2-Gal4* [S13]). For genetic interaction analysis, the following were made: *w; UAS-Him/UAS-Him; gro*^{BX22}*/ TM3, lacZ*, *w; twi-Gal4/ twi-Gal4; gro*^{E48}*/ TM3, lacZ*, and *UAS-Him/UAS-Him*; *UAS-mef2/ UAS-mef2*. The first two of these stocks were crossed to analyze *Him* overexpression when *gro* function is reduced.

UAS-Him RNAi Analysis

The knockdown of *Him* expression was assessed first by in situ hybridization. Figure S2 shows a representative example. The knockdown was then quantitated by qRT-PCR of late stage 12/early stage 13 embryos (9 hr 10 min–9 hr 40 min AEL) as follows. RNA was isolated from three independent collections of >150 individually staged embryos from both wild-type and *UAS-Him RNAi*. cDNA generated with oligo dT was used to program a standard SYBR green q-PCR reaction. The (+) *Him* PCR primer spanned the single *Him* intron; the (−) strand primer was in the 3′UTR (sequences available on request). Each of the three biological replicates was assayed in triplicate for *Him* and the reference gene *rp49*. The expression of *Him* mRNA in *Him* RNAi relative to the wild-type was calculated with the $2^{-\Delta\Delta C(T)}$ method [S14]. The average reduction in the *Him* RNAi condition was to 44% of the wild-type *Him* RNA levels.

Hatching and Survival Assay

Three hundred developing embryos were aligned on apple-juice agar plates at 18°C, and the number of newly hatched larvae was scored. Surviving third instar larvae were put in tubes and allowed to develop until adulthood when the number of eclosing flies was scored.

Muscle Phenotype Analysis

The somatic-muscle phenotype was scored through examination of the muscle pattern. Each of the 30 muscles per abdominal hemisegment was analyzed systematically in three hemisegments (A2 to A4) for any differences compared to the wild-type. For description of the RNAi phenotype, 101 embryos were analyzed. For assessment of the genetic interaction between *UAS-Him* and *gro* or *UAS-mef2*, at least 45 embryos of each genotype were analyzed. The number of wild-type muscles was recorded for each embryo, and the mean was determined and then compared between *UAS-Him* alone and *UAS-Him* plus either *gro* or *UAS-mef2*. The significance was assessed with a two-sample t test.

Him Sequence Analysis

Analysis of the predicted Him protein sequence with a range of programs did not provide any evidence for an HLH domain, although they did detect the HLH domain of Hairy, a *Drosophila* bHLH protein that contains a WRPW motif. These programs included the following: Prosite (http://expasy.org/prosite/), InterProScan (http://www.ebi.ac.uk/interproscan/), SMART (http://smart.embl-heidelberg.de/), Blocks (http://blocks.fhcrc.org/), Pfam (http://www.sanger.ac.uk/software/pfam/), and The Conserved Domain Database at NCBI (http://www.ncbi.nlm.nih.gov/entrez/query.fcgi?db=cdd). The PSORTII program (http://psort.hgc.jp/) identified a putative bipartite NLS in the predicted Him protein sequence.

In Vitro Protein-Protein Interactions

The *GST-Him* and *GST-HimΔWRPW* constructs were generated with the pET-3a vector (Novagen), in which we removed the T7-Tag and replaced it with GST to create GST-pET-3a. *Him* and *Him-ΔWRPW* DNA fragments were generated by PCR from *Him* cDNA, cloned into pGEM-T (Promega), and subcloned into GST-pET-3a. In vitro-translated [^{35}S]-Met-labeled Gro was produced, GST-fusion proteins were expressed and purified, and in vitro binding assays were undertaken as described [S15]. Analysis was by SDS PAGE, and radiolabeled Gro was visualized by autoradiography.

S2 Expression Assay

Mef2 activity was assayed with a *mIR-1* luciferase reporter after transfection into *Drosophila* S2 cells as described previously [S16].

Supplemental References

S1. Taylor, M.V. (2000). A novel Drosophila, mef2-regulated muscle gene isolated in a subtractive hybridisation-based molecular screen using small amounts of zygotic mutant RNA. Dev. Biol. *220*, 37–52.

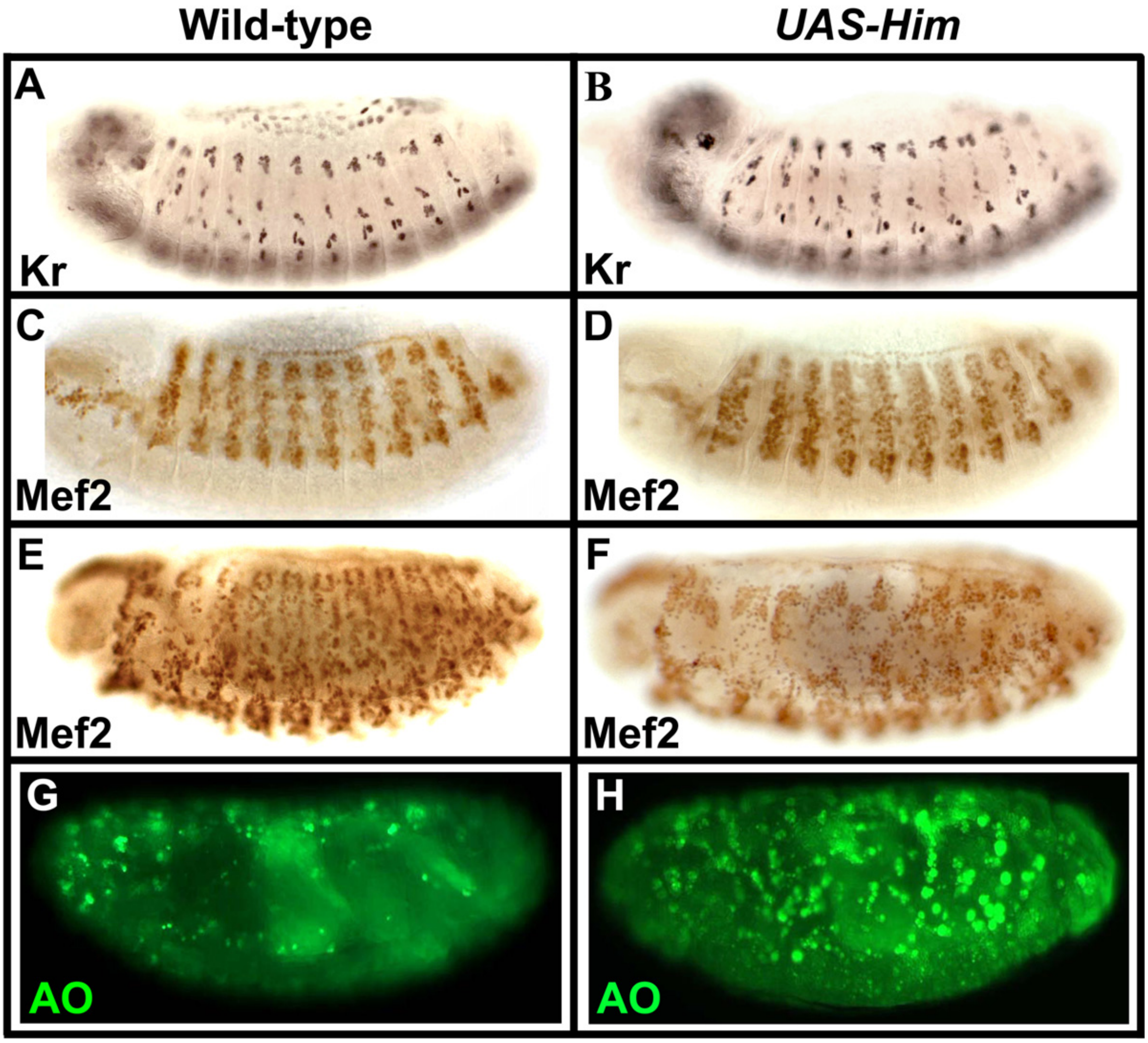

Figure S1. *Him* Affects the Differentiation Phase of Muscle Development

UAS-Him expression was driven in the developing mesoderm by *twi-Gal4;twi-Gal4* at 25°C (B, D, F, and H) and compared with the wild-type (A, C, E, and G). When *Him* is overexpressed, muscle development proceeds similarly to the wild-type up to stage 13, as shown by immunostaining for the founder cell marker Kruppel (A and B) and for Mef2 (C and D). However, immunostaining for Mef2 shows that differentiation is dramatically affected by *Him* at stage 15 (E and F). The finding that acridine orange (AO) reveals an increase in cell death at stage 16 (G and H) suggests that cells that fail to differentiate into muscle die. At stage 17, *Him* overexpression results in a dramatic reduction in Myosin-expressing cells (Figure 1). In each case, representative examples of the phenotype are shown.

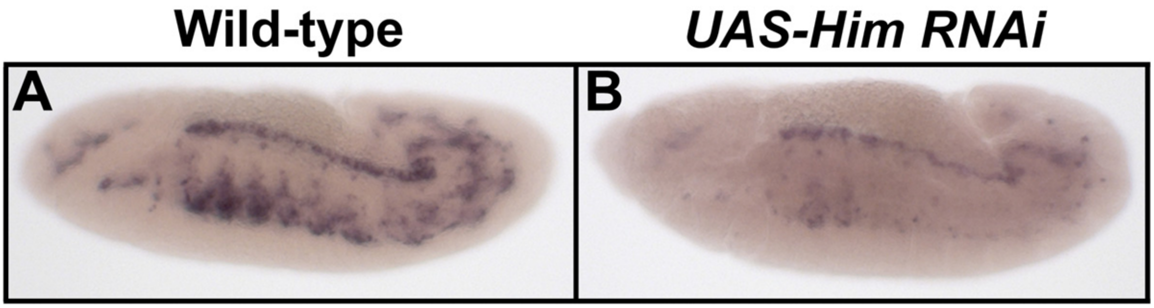

Figure S2. *Him RNAi* Knocks Down *Him* Expression

In situ hybridization for *Him* RNA in stage 12 embryos in the wild-type (A) and *UAS-Him RNAi* driven by *twi-Gal4;twi-Gal4* at 25°C (B) shows that *Him RNAi* knocks down the expression of *Him*. Representative examples of each condition are shown.

S2. Sullivan, W., Ashburner, M., and Hawley, S. (2000). *Drosophila* Protocols (Cold Springs Harbor, NY: Cold Spring Harbor Laboratory Press).

S3. Ruiz-Gomez, M., and Ghysen, A. (1993). The expression and role of a proneural gene, achaete, in the development of the larval nervous system of Drosophila. EMBO J. *12*, 1121–1130.

S4. Kaltschmidt, J.A., Lawrence, N., Morel, V., Balayo, T., Fernandez, B.G., Pelissier, A., Jacinto, A., and Martinez Arias, A. (2002). Planar polarity and actin dynamics in the epidermis of Drosophila. Nat. Cell Biol. *4*, 937–944.

S5. Brand, A.H., and Perrimon, N. (1993). Targeted gene expression as a means of altering cell fates and generating dominant phenotypes. Development *118*, 401–415.

S6. Reichhart, J.M., Ligoxygakis, P., Naitza, S., Woerfel, G., Imler, J.L., and Gubb, D. (2002). Splice-activated UAS hairpin vector gives complete RNAi knockout of single or double target transcripts in Drosophila melanogaster. Genesis *34*, 160–164.

S7. Kaltschmidt, J.A., Davidson, C.M., Brown, N.H., and Brand, A.H. (2000). Rotation and asymmetry of the mitotic spindle direct asymmetric cell division in the developing central nervous system. Nat. Cell Biol. *2*, 7–12.

S8. Rubin, G.M., and Spradling, A.C. (1982). Genetic transformation of Drosophila with transposable element vectors. Science *218*, 348–353.

S9. Bour, B.A., Obrien, M.A., Lockwood, W.L., Goldstein, E.S., Bodmer, R., Taghert, P.H., Abmayr, S.M., and Nguyen, H.T. (1995). Drosophila Mef2, a transcription factor that is essential for myogenesis. Genes Dev. *9*, 730–741.

S10. Ranganayakulu, G., Zhao, B., Dokidis, A., Molkentin, J.D., Olson, E.N., and Schulz, R.A. (1995). A series of mutations in the D-Mef2 transcription factor reveal multiple functions in larval and adult myogenesis in Drosophila. Dev. Biol. *171*, 169–181.

S11. Preiss, A., Hartley, D.A., and Artavanis-Tsakonas, S. (1988). The molecular genetics of Enhancer of split, a gene required for embryonic neural development in Drosophila. EMBO J. *7*, 3917–3927.

S12. Baylies, M.K., and Bate, M. (1996). twist: A myogenic switch in Drosophila. Science *272*, 1481–1484.

S13. Carmena, A., Buff, E., Halfon, M.S., Gisselbrecht, S., Jimenez, F., Baylies, M.K., and Michelson, A.M. (2002). Reciprocal regulatory interactions between the Notch and Ras signaling pathways in the Drosophila embryonic mesoderm. Dev. Biol. *244*, 226–242.

S14. Livak, K.J., and Schmittgen, T.D. (2001). Analysis of relative gene expression data using real-time quantitative PCR and the 2(-Delta Delta C(T)) method. Methods *25*, 402–408.

S15. Jennings, B.H., Pickles, L.M., Wainwright, S.M., Roe, S.M., Pearl, L.H., and Ish-Horowicz, D. (2006). Molecular recognition of transcriptional repressor motifs by the WD domain of the Groucho/TLE corepressor. Mol. Cell *22*, 645–655.

S16. Kwon, C., Han, Z., Olson, E.N., and Srivastava, D. (2005). MicroRNA1 influences cardiac differentiation in Drosophila and regulates Notch signaling. Proc. Natl. Acad. Sci. USA *102*, 18986–18991.
